# General anaesthesia in end‐of‐life care: extending the indications for anaesthesia beyond surgery

**DOI:** 10.1111/anae.15459

**Published:** 2021-04-20

**Authors:** A. Takla, J. Savulescu, D. J. C. Wilkinson, J. J. Pandit

**Affiliations:** ^1^ Uehiro Centre for Practical Ethics Faculty of Philosophy University of Oxford Oxford UK; ^2^ Uehiro Centre for Practical Ethics Faculty of Philosophy University of Oxford Oxford UK; ^3^ Murdoch Children’s Research Institute Royal Children’s Hospital Melbourne Australia; ^4^ Uehiro Centre for Practical Ethics Faculty of Philosophy University of Oxford Oxford UK; ^5^ Murdoch Children’s Research Institute Royal Children’s Hospital Melbourne Australia; ^6^ Department of Neonatology Oxford University Hospitals NHS Foundation Trust Oxford UK; ^7^ Nuffield Department of Anaesthetics Oxford University Hospitals NHS Foundation Trust Oxford UK; ^8^ University of Oxford Oxford UK

**Keywords:** palliative care, dying, general anaesthesia, ethics, sedation

## Abstract

In this article, we describe an extension of general anaesthesia – beyond facilitating surgery – to the relief of suffering during dying. Some refractory symptoms at the end of life (pain, delirium, distress, dyspnoea) might be managed by analgesia, but in high doses, adverse effects (e.g. respiratory depression) can hasten death. Sedation may be needed for agitation or distress and can be administered as continuous deep sedation (also referred to as terminal or palliative sedation) generally using benzodiazepines. However, for some patients these interventions are not enough, and others may express a clear desire to be completely unconscious as they die. We summarise the historical background of an established practice that we refer to as ‘general anaesthesia in end‐of‐life care’. We discuss its contexts and some ethical and legal issues that it raises, arguing that these are largely similar issues to those already raised by continuous deep sedation. To be a valid option, general anaesthesia in end‐of‐life care will require a clear multidisciplinary framework and consensus practice guidelines. We see these as an impending development for which the specialty should prepare. General anaesthesia in end‐of‐life care raises an important debate about the possible role of anaesthesia in the relief of suffering beyond the context of surgical/diagnostic interventions.

## Introduction

There is a potential role for general anaesthesia in end‐of‐life care, something which has been described previously but largely overlooked. We will start by reviewing the current place of sedation in end‐of‐life care and show how general anaesthesia is an extension of existing practices. However, introducing general anaesthesia in end‐of‐life care also requires an examination of the associated ethical, medicolegal and practical implications. We stress that general anaesthesia in end‐of‐life care is not a form of assisted dying or euthanasia [1, 2], which are illegal in the UK.

## The current role of sedation in end‐of‐life care

Many symptoms (delirium, dyspnoea and pain) can become resistant to treatment at the end of life [3]. Up to 84% of patients are reported to require opioids [4, 5]. Additional interventions familiar to anaesthetists include the use of non‐opiate medications, epidural and intrathecal regional anaesthesia, local anaesthesia infiltration or blocks, and even acupuncture or nerve stimulation [6]. Some patients may need antidepressants (which have analgesic properties in the context of chronic pain) or benzodiazepines to aid sleep and antipsychotics to manage delirium [7]. The palliative care formulary lists agents used at the end of life and examples of approaches used can be found in national and individual hospital guidelines [8, 9].

Patients who are dying are particularly vulnerable to the cardiorespiratory adverse effects of some agents (e.g. opioids, midazolam, phenobarbital, ketamine). While there is little evidence that these occur with judicious use [5, 10], it is important that fear of such adverse effects does not lead to restrictive treatment practices. The so‐called ‘doctrine of double effect’ legally and ethically permits the intentional relief of suffering, even where it is foreseen that there is a significant risk of adverse effects that might hasten death [11].

The specific need for sedation over and above opioid analgesia in end‐of‐life care is variable, reported as being between 3% and 51% in different case series [12]. This may reflect differences in local practices [12, 13] and the host of different terms used, including: ‘palliative sedation’, ‘terminal sedation’ or ‘continuous deep sedation’ [14]. In this paper, we will use the latter term. Current UK guidance from the National Institute for Health and Care Excellence (NICE), supported by the Royal College of Anaesthetists, makes no specific recommendations about sedation, and instead constitutes broad principles underlying the management of end‐of‐life care [15].

Regardless of the intended level of sedation, what is actually experienced by patients receiving continuous deep sedation varies [16]. Some patients are lightly sedated, others are mostly unconscious, stirring intermittently or only in response to vigorous stimulation, while others are almost anaesthetised [16, 17, 18, 19, 20]. In part, this is because a variety of techniques, drugs and doses are used. Benzodiazepines are employed commonly (usually midazolam), but other drugs, including barbiturates (such as phenobarbital), are also prescribed. Moreover, ‘sedation’ encompasses a continuous spectrum, with anxiolysis in an awake patient at one extreme and a state approaching general anaesthesia at the other. There is no clear clinical or neuroscientific cut‐off between these states of loss of consciousness, and the term ‘dysanaesthesia’ has been used to describe one such ‘in‐between’ state [21]. Sedation typically means that verbal contact with the patient is retained; that is, they are co‐operative when aroused. By contrast, general anaesthesia refers to a state where the patient is unarousable even to painful stimuli [22].

## Patients’ desire to be unconscious

Patients have different wishes in relation to suffering and its alleviation. Sigmund Freud, when dying of throat cancer, preferred to have a clear head and ‘*think in torment’* [23], refusing analgesia or sedation. Others may have the opposite preference. In a recent survey, 88% of a large online survey of the UK public expressed a wish to have the option of unconsciousness at the end of life [24].

The desire to be unconscious as a means of eliminating the experience of physical or mental suffering is understandable. Unconsciousness achieves the highest probability of being unaware that the body is going through an adverse process, since even that awareness may be psychologically traumatic to some. This is recognised in other very different contexts. For example, requests by healthy patients for general anaesthesia for minor surgery (e.g. dental extraction) are viewed as entirely acceptable by anaesthetists and by society, even when it is clear that mild sedation (or none at all) would be sufficient to avoid pain, and that anaesthesia poses a greater risk of adverse events. This is out of respect for patients’ autonomy. At the end of life, patients may experience feelings of sadness, fear, anxiety, loneliness, vulnerability, embarrassment and loss of dignity [25], in addition to pain, suffocation and other suffering, not all of which may be relieved by targeted treatments.

## Patients’ expectations of unconsciousness

We now know that when many patients agree to ‘sedation’, they are in fact expecting to be unconscious. The proportion of patients who report being ‘accidentally aware’ during surgical interventions was higher when patients were sedated than when they received general anaesthesia [26]. Moreover, it has become apparent that patients appearing to be unconscious in several situations may in fact not be. Patients who are critically ill, or those with severe head injury diagnosed as being in deep coma, have reported recall of events in critical care [26]. This also appears to be the case with current terminal sedation techniques, where bispectral index scores can remain > 60 in patients who appeared unconscious [27]. In patients diagnosed as being in a vegetative state, studies show that some apparently unconscious patients can reliably generate appropriate electroencephalographic responses to stimuli [28] or commands [29] and in some cases communicate ‘yes’ or ‘no’ answers (as evidenced by functional magnetic resonance imaging patterns [30]). This raises the possibility that some patients who are dying may be suffering when otherwise believed not to be.

## General anaesthesia in end‐of‐life care in practice: rediscovering the Moyle protocol

Perhaps the first description of using general anaesthesia in end‐of‐life care was in 1995 by John Moyle, a consultant anaesthetist and palliative care physician. Moyle recognised the limitations of conventional approaches as discussed above (J. Moyle, personal communication). Moyle developed a protocol for infusing the then relatively new anaesthetic agent propofol and described its use in two patients, who died peacefully after 4 and 9 days of continuous infusion [31]. In the same year, a similar case and protocol was reported in Italy [32]. Since then, propofol infusions in end‐of‐life care have been described regularly, albeit infrequently, in the palliative care literature [33, 34, 35].

Moyle anticipated some technical challenges with his protocol. Intravenous induction of anaesthesia by bolus invariably results in potent respiratory and cardiovascular depression. The former is associated with apnoea and upper airway collapse. If the anaesthetist does not provide airway support, induction of anaesthesia itself becomes life terminating. These respiratory adverse effects do not occur as rapidly as with benzodiazepine sedatives like midazolam, and these agents can be reversed with flumazenil. Similarly, any excessive respiratory depression by opiates can be reversed promptly using naloxone. Hypotension and cardio‐depressive effects might also hasten the process of dying if not managed using appropriate drugs or intravenous fluids.

To avoid these complications, Moyle and others recommended very slow intravenous infusion by a pump at a carefully titrated dose (e.g. just 5 mg.h^‐1^ vs. the 100–200 mg typically used as a bolus) [31]. The depth of anaesthesia achieved was inadequate for a surgical procedure, but was ideal for an undisturbed dying patient. In a case series of 22 patients from a Swedish specialist palliative care unit, propofol infusions were provided in this way for continuous deep sedation for between 1 and 14 days (median 5 days) [34]. Notably, the protocol developed and used by Moyle did not require the level of continuous patient monitoring that might be necessary during surgical general anaesthesia [36].

This is not to imply that propofol is an anaesthetic whereas other drugs like benzodiazepines are not. Any suitable sedative given in sufficient doses will achieve unresponsiveness to painful stimulation. Rather, the distinctive element of this approach is the intended purpose of achieving general anaesthesia, rather than sedation.

## Updating the Moyle protocol for anaesthesia in end‐of‐life care

Since Moyle’s description, there have been about 25 publications, case series and reviews of intended general anaesthesia using propofol infusions at the end of life. One international perspective is relevant. Since 2016, terminally ill patients in France have a legal right to unconsciousness once it is deemed they are dying, sustained until the point of death [37]. This has been labelled the ‘French exception’ emphasising the unique character of the French legal code in this regard [37]. As a result, the Haute Autorite de Santé (the body responsible for setting medical standards) has issued clear guidance on how ‘end of life’ is to be defined, what protective steps should be taken to achieve medical consensus that sedation is desirable and the means of administering it [38]. These guidelines refer primarily to the use of intravenous midazolam but mention that propofol may also be used with expert anaesthetic input [38]. This is, therefore, a strong official implication that anaesthetists might, or should, be directly involved. The guidelines do not offer technical details, but it is theoretically straightforward to update Moyle’s established protocol to the modern era, where propofol target‐controlled infusions are commonly used in anaesthesia.

Ultra‐slow intravenous induction is now described in at least two contexts. One is to mimic an inhalational induction of anaesthesia (spontaneous respiration using intravenous anaesthesia, STRIVE) [39]. The second is in basic neuroscience experimentation, where ultra‐slow propofol target‐controlled infusions (0.2 µg.ml^‐1^ increments over 50 min) are titrated to maintain spontaneous respiration while volunteers are lying in a functional MRI scanner [40]. Interestingly, these experiments have revealed brain activity patterns of ‘thalamic isolation’, temporally associated with the behavioural effect of the subjects becoming inattentive or disinterested in their surroundings, consistent with the notion of dysanaesthesia. Specific adaptations of methods like these have been discussed by Bodnar [41]; further technical details are outside the scope of this article but should be self‐evident to most anaesthetists. Table 1 indicates the potential place of general anaesthesia in end‐of‐life care alongside other more standard options and makes a distinction between intended sedation vs. intended general anaesthesia.

## Ethical issues

### Patient autonomy

One key argument in favour of anaesthesia in end‐of‐life care revolves around the significance of patient preference and their personal concept of a good death [11]. General anaesthesia in end‐of‐life care is not intended to replace analgesia or sedation for symptom control. However, it should be an additional option for those who want more reliable or complete loss of consciousness than can be offered currently. Some patients may choose general anaesthesia in end‐of‐life care only once these other modalities have been attempted and, in their experience, failed. However, others may choose it as a first choice once death is imminent. Others, perhaps the majority, might not require or request it at all.

Respecting patient autonomy in this way does not imply that the final decision about therapy belongs solely to the patient, nor that general anaesthesia in end‐of‐life care should be available on request to any patient at any time. We set out some proposed conditions for the institution of general anaesthesia in end‐of‐life care below, based on the French experience. Instead, decision‐making should, as ever, be a dynamic process between the patient and their families, and the medical staff (including physician, nurses and other carers) which engages both clinical expertise and patient values and preferences.

### Offering effective treatments

It is a fundamental ethical principle in medicine that doctors should promote and act in patients’ best interests (beneficence), and by extension, offer them the therapy option most likely to achieve the desired outcome [42]. If a competent, dying patient wishes to be unconscious (and not just sedated) as a specifically stated aim to help relieve their suffering, then general anaesthesia in end‐of‐life care provides the greatest assurance of meeting this wish and is, therefore, in the best interests of the patient.

Studies of surgical anaesthesia indicate that patients unambiguously requesting general anaesthesia are not satisfied (and indeed, may suffer long‐term psychological distress) if sedation is provided instead [26]. If sedation at the end of life leaves the patient in a semi‐conscious state but unable to communicate meaningfully, there is concern they would have severely aversive experiences similar to, or even worse than, patients who have reported distressing ‘accidental awareness’ during surgery. We now know that unconsciousness is better achieved with intended general anaesthesia. In the absence of neuromuscular blocking drugs, the incidence of accidental awareness is in the order of 1 in 136,000 [26].

### Informed consent

It is important that patients are informed of all the options available to them in relation to the relief of suffering at the end of life: analgesia, sedation and, potentially now, anaesthesia. The risks and benefits of each should be explained. Patients should be free to choose the option, or sequence of options or combination of options, which best meet their values (autonomy), consistent with distributive justice.

However, general anaesthesia in end‐of‐life care creates a situation in which autonomy is more reliably and irreversibly lost. Patients will not be able to change their minds or revise their decision. This is not a problem unique to general anaesthesia in end‐of‐life care, as it is already potentially the case with continuous deep sedation. It is resolved through careful prior consent, where a patient choosing either general anaesthesia in end‐of‐life care or continuous deep sedation needs to be aware in advance that their decision is likely irreversible. This is not something ever made explicit in consent for general anaesthesia in surgery but it is self‐evident that a patient cannot change their mind after induction of anaesthesia. Because anaesthetists are trained to obtain consent for anaesthesia from which the patient is expected to recover, the very different aim of general anaesthesia in end‐of‐life care may require additional training. This is not addressed in existing consent guidelines [43], and may necessitate the involvement of other specialties. Consent should also include the possibility that the patient awakens during extended general anaesthesia in end‐of‐life care (e.g. due to interruption of infusion, altered sensitivity to agent, prolonged process of dying, etc.).

### Avoidance of hastening death

There may be a concern (in our view, misplaced) that anaesthesia in end‐of‐life care will, unintentionally, hasten death [44]. There is no statistically significant difference in mean survival time between patients at the end of life who receive continuous deep sedation and those who do not [45, 46]. Data are limited for general anaesthesia in end‐of‐life care but a propofol infusion has been continued for up to 14 days without suppressing spontaneous breathing [34], and in another study in paediatric oncology patients, propofol was continued for up to 10 days [34, 35]; these do not appear foreshortened times to death. None of the studies describing the technique suggest death was hastened [31, 32, 34, 35].

In law, the possibility of medical interventions at the end of life unintentionally hastening death is covered by the ‘doctrine of double effect’, as discussed above. If it is wrongly assumed that general anaesthesia in end‐of‐life care (being anaesthesia) is inherently and prohibitively riskier than continuous deep sedation (which is sedation), then there may arise a concern that this doctrine may not be applied by courts or regulators because they judge there to be, in their minds, a safer alternative. However, there is no empirical evidence in this or any other context that deep sedation is inherently safer than anaesthesia.

To minimise concerns about hastening death, one practical option for anaesthesia in end‐of‐life care would be to limit its application to patients who are predicted to die within a period of up to 14 days, as suggested in the French guidance [37, 38]. The rationale for this timeframe is that beyond 14 days, there may be concerns that malnutrition would constitute a foreseeable, independent and avoidable contributor to death. Moreover, with general anaesthesia in end‐of‐life care, the patient is unable to chew and swallow, and even nasogastric tube feeding may lead to regurgitation and aspiration, precipitating respiratory failure. Whether intravenous hydration should be continued or avoided, is a matter of separate clinical judgement which should involve consulting the patient (if they can participate in decision‐making), their surrogate or reviewing an advance directive (if available).

Setting an upper limit of 14 days for the administration of anaesthesia in end‐of‐life care might, however, raise another dilemma if the patient does not die within that time frame. One option would be to awaken and reassess the patient, their wishes, and their medical status. Dying is a very uncertain and unpredictable process. However, this is not a unique problem to anaesthesia in end‐of‐life care and could be addressed in the same way as other existing interventions (e.g. continuous deep sedation) and decisions in palliative care where patients survive for longer than anticipated initially.

### Fundamental human rights

Three relevant fundamental rights are already established in law, or by custom and practice: patients with a terminal illness have a right to access palliative care, which includes a right to analgesia and anxiolysis including sedation [15] and (in France) a right to unconsciousness [37]; patients have a right to refuse life‐prolonging treatment (including artificial nutrition and hydration) at any time [47]; and patients have a right to choose general anaesthesia over clinically effective alternatives when they undergo interventions that might confer suffering (commonly surgery or diagnostic procedures). We can then draw together these individual rights to justify the use of general anaesthesia in end‐of‐life care to manage suffering. Terminally ill patients should have a right to the option of anaesthesia as they approach the end of their life.

It is important to stress that while general anaesthesia in end‐of‐life care should be an option, it is an open question how many patients would choose it. Other than the evidence from the palliative care literature that describes anaesthesia in end‐of‐life care, we cannot estimate the size of the unmet need. However, even if only a few would choose it, that alone does not provide an ethical objection.

### Ethics of resource allocation and distributive justice

The practice of general anaesthesia in end‐of‐life care prima facie requires expert anaesthetic input, an additional resource requirement to more conventional forms of end‐of‐life care. Additional support may also be necessary for training. This might generate opposing ethical dilemmas. If general anaesthesia in end‐of‐life care is ethically justified, then it is important to allocate resources to provide it, which might then reduce anaesthesia resources to support surgical services. Conversely, if anaesthesia capacity is limited by commitments to surgery, then terminal patients may be denied care to which they have a right. The costs of providing it as an option would need to be factored into planning models for end‐of‐life care at a national level.

## Legal perspectives

### Patient perspective

If the option of general anaesthesia in end‐of‐life care is not widely known or used, then patients may not be offered it in the first place. Therefore, professionals need to be aware of it, and also be assured of a supportive professional framework. Regardless, a motivated, well‐informed patient could request anaesthesia in end‐of‐life care and this may become a driver for change. The ‘French exception’ is clearly consistent with the European Convention on Human Rights and does not conflict with any other laws prohibiting euthanasia in France. This cannot be extrapolated automatically to the UK, but the principles could be used by a UK petitioner (patient) to assert an equivalent ‘right to unconsciousness’. At the very least, it would be fascinating to observe if or how a UK Supreme Court that requires doctors to share with patients the risks and benefits of all treatment options that are ‘materially important to them’ (under the ‘Montgomery principle’) would decide such a case [48].

### Physician perspective

In practice, any future request for general anaesthesia in end‐of‐life care in the UK would be expected to arise from a hospice patient’s supervising palliative care physician or general practitioner, directed to an anaesthetist. Regardless of the willingness or competence of an anaesthetist to provide this service, there would be practical issues in the management of drugs and devices (e.g. where they would be obtained from, maintained, etc.).

Administering general anaesthesia as part of end‐of‐life care is not expressly prohibited by UK law, which does not stipulate limits on the purposes for which general anaesthesia should be administered. Nevertheless, it will be a concern in the minds of many physicians that any interventions they make at the end of life may be misconstrued, with the threat of investigation, sanction or serious criminal prosecution. As noted above, the ‘doctrine of double effect’ is potentially protective, and the related practice of continuous deep sedation is well established. Since general anaesthesia in end‐of‐life care has already been described in the UK and several other European countries, this literature base represents a ‘responsible body of medical opinion’ and, as suggested above, a reasonable one. However, there may be a practical limitation to using the existing literature base in any defence as it might be difficult to locate a UK physician actually practising the technique to support it. Moreover, any individual administering anaesthesia in end‐of‐life care might need to demonstrate how it falls within their own normal range of clinical practice and experience.

## Conclusion

In summary, we have described a potential role for general anaesthesia in end‐of‐life care as something de facto available to UK patients since the 1990s. There is a strong ethical case for making this option more widely available. Describing general anaesthesia in end‐of‐life care the way we do does not imply that existing palliative care practice is deficient. Indeed, general anaesthesia in end‐of‐life care might be requested by, or suitable for, very few patients. However, the number of patients involved should not alone determine whether this issue is regarded as ethically important. Even if complete unconsciousness is desired by only a few patients, there is a moral imperative upon national anaesthesia, palliative care and nursing organisations to prepare for the possibility that general anaesthesia in end‐of‐life care may be requested by some patients, and to work collaboratively to develop clear protocols to address the practical, ethical and medicolegal issues arising.

Ours is a theoretical exploration of what we anticipate may become a wider phenomenon, observing recent developments in Europe. Early descriptions of this extended use of general anaesthesia using propofol in terminal care by multidisciplinary teams are appearing in the anaesthetic literature [49]. Mercadante and Giarratano have outlined how the skills of anaesthetists readily map onto many interventions at the end of life [50]. Implementing any practical guidance will require collaboration with palliative care physicians, nurses, patient groups and others. Moreover, a fundamental challenge for national anaesthetic societies will be to accept the novel notion that administration of general anaesthesia has a place in the relief of suffering generally, not just confined to facilitating surgery, critical care or diagnostic interventions. The speciality already has a self‐declared mission to extend the role of anaesthetists beyond the operating theatre in its strategy of championing ‘peri‐operative medicine’ [51]. Our redefinition of the scope and reach of general anaesthesia through end‐of‐life care, radical though it is, would be entirely in line with that philosophy, recognising that anaesthetists have skills that can help alleviate suffering for the dying patient.

**Table 1:** Summary of place of different treatment options during end‐of‐life care. The first three rows summarise existing practice (see: https://www.palliativecareguidelines.scot.nhs.uk); the last row indicates the potential role for general anaesthesia in end‐of‐life care. All regimens marked * may produce an unresponsive state akin to general anaesthesia, with sufficient dosage of drug(s) or their synergistic combinations.

Intervention option	Intended effect; route of administration; and when commenced in course of terminal illness	Effect on patient	Limitations/adverse effects with increasing doses
Opiate analgesia*	Pain relief; oral or parenteral administration (but rarely intravenous); can be commenced at any point in the illness	Analgesia, euphoria but also increased somnolence, reduced ability to concentrate	Respiratory depression, associated with unconsciousness
Sedation with benzodiazepines (or barbiturates)*	To assist with anxiety and aid sleep and anti‐depressive effects; oral administration; can be commenced at any point in the illness	Anxiolysis, increased somnolence; may reduce or prevent the ability to communicate clearly	Loss of verbal contact (thus equating to a state of general anaesthesia); unconsciousness; respiratory depression
Continuous deep sedation (with midazolam)*	Unconsciousness, continuous intravenous infusion; under existing protocols commenced within 2 weeks of predicted death	The patient is unresponsive to verbal command and apparently unaware of surroundings; however, strong stimuli may rouse the patient	When dose and concentrations are controlled carefully, there is no evidence that continuous deep sedation hastens death; however, patients may be aware and left unable to communicate. Protective reflexes like cough are obtunded, so aspiration is a risk
General anaesthesia in end‐of‐life care (with propofol)	Unconsciousness, continuous intravenous infusion; to be consistent with continuous deep sedation protocols, to be commenced within 2 weeks of predicted death	The patient is unresponsive to verbal command or strong stimuli and better assurance of unawareness of surroundings and relief of suffering vs. continuous deep sedation	When dose and concentrations are controlled carefully, there is no evidence that general anaesthesia in end‐of‐life care hastens death; however, care needed at induction where profound respiratory or cardiovascular depression can occur unless very slow infusion rates used. Protective reflexes like cough are abolished, so aspiration is a risk
